# Fibroblasts from long-lived species of mammals and birds show delayed, but prolonged, phosphorylation of ERK

**DOI:** 10.1111/acel.12172

**Published:** 2013-11-13

**Authors:** Najoua Elbourkadi, Steven N Austad, Richard A Miller

**Affiliations:** 1 Department of Pathology and Geriatrics Center, University of MichiganAnn Arbor, MI, 48109, USA; 2 Barshop Center, University of Texas Health Science Center15355 Lambda Drive, San Antonio, TX, 78245-3207, USA

**Keywords:** birds, evolution, longevity regulation, life span, mammals, oxidative stress, signalling

## Abstract

Fibroblasts from long-lived mutant mice show diminished phosphorylation of the stress-activated protein kinases ERK1/2 after exposure to peroxide, cadmium, or paraquat. We have now evaluated the kinetics of ERK phosphorylation in fibroblasts from long-lived and short-lived species of mammals and birds in response to stress by cadmium or hydrogen peroxide. Fibroblasts from the shorter-lived species of rodents and birds showed rapid induction of ERK phosphorylation, with a decline to basal level within 60 min. In contrast, cells from longer-lived species showed slower and more prolonged activation of ERK phosphorylation. These results suggest that fibroblasts from long-lived species may be less susceptible to the early phases of damage from cadmium or peroxide and suggest that altered kinetics of ERK activity may contribute to their stress resistance properties.

## Introduction

The molecular basis for the great diversity in aging rate and maximum longevity among species of mammals and birds is not yet well understood (Austad, 2009). Changes in genotype (Brown-Borg *et al*., 1996; Flurkey *et al*., 2001), diet (Orentreich *et al*., 1993; Weindruch & Sohal, 1997), drug exposure (Harrison *et al*., 2009), or breeding opportunities (Dammann *et al*., 2011) can increase maximum lifespan by as much as about 50% within a given species of mammal, but evolutionary processes can lead to differences of nearly 100-fold between the shortest-lived and longest-lived species of mammals, with a range of about tenfold even within some of the mammalian orders. Long-lived species have evolved many times within the mammalian and avian radiations, but it is not yet clear to what extent the mechanisms that contribute to exceptionally long lifespan in one species resemble those with slow aging in distantly related clades.

There is some evidence that cultured cells from long-lived organisms may differ systematically from cells derived from shorter-lived species. Fibroblasts cultured from longer-lived species of mammals are relatively resistant to death induced by a variety of oxidative toxins (Kapahi *et al*., 1999). Mouse cell lines are more susceptible than human fibroblasts to oxygen-mediated mutation and growth crisis (Busuttil *et al*., 2003; Parrinello *et al*., 2003), but do not show telomere-based clonal senescence when cultured at physiologically low oxygen tensions. A study of fibroblasts from 15 rodent species showed (Seluanov *et al*., 2008) that they could be divided into three classes based on differences in tumor-suppressive mechanisms, with telomere-based growth control characteristic of the larger species. Among the rodent species of small body size, longer lifespan was associated with a slower rate of *in vitro* cell proliferation. Work in our own laboratory has shown (Harper *et al*., 2007) that fibroblasts from longer-lived rodent species are resistant, in culture, to death induced by exposure to cadmium and H_2_O_2_, with similar but nonsignificant trends in cells exposed to heat (*P* = 0.053) or to the DNA-alkylating agent MMS (methyl methanesulfonate, *P* = 0.08). Fibroblasts from relatively long-lived species of birds were, similarly, found to be resistant to death induced by cadmium, paraquat, and MMS, with a suggestive trend (*P* = 0.07) in resistance to H_2_O_2_. Interestingly, bird fibroblasts were more resistant than rodent fibroblasts to death induced by cadmium, paraquat, H_2_O_2_, MMS, and ultraviolet (UV) light (Harper *et al*., 2011), consistent with the relatively longer lifespan of birds compared with rodent species of similar body size (Holmes *et al*., 2001). These findings parallel the observation that fibroblasts from long-lived mutant mouse stocks, including the Snell dwarf, Ames dwarf, and growth hormone receptor knockout mice, are resistant in culture to death induced by H_2_O_2_, paraquat, cadmium, heat, UV light, MMS, and arsenite (Murakami *et al*., 2003; Salmon *et al*., 2005; Leiser & Miller, 2009), suggesting that at least some of the properties seen in cells from long-lived mutant mice might be related to those through which evolutionary pressures mold life history and aging rates across species.

Responses of cells to stress are modulated, in part, by enzymes in the mitogen-activated protein kinase (MAPK) signaling pathway. This group, which includes sets of ERK, JNK, and p38 kinases (Roux & Blenis, 2004), consists of ubiquitously expressed signaling proteins that are involved in many facets of cellular regulation, including proliferation, differentiation, adaptive responses to hormones, nutrient availability, and injurious stresses. Many of these kinases have been reported to play a role in various diseases and have been implicated in cell injury (Chang & Karin, 2001; Narasimhan *et al*., 2009). The extracellular-signal-regulated protein kinase ERK typically exists in two isoforms, ERK1 and ERK2, also called p42 and p44 kinases, and serves as one of the major transducers of extracellular signals connecting the membrane-bound receptors to changes in cellular function (Costa *et al*., 2006). In mammals, ERK1 and ERK2 have 83% amino acid identity and are expressed in varying amounts and proportions in all tissues (Chen *et al*., 2001; Roux & Blenis, 2004). In quiescent cells, ERK1/2 is typically dispersed throughout the cell, but a significant population of ERK1/2 accumulates in the nucleus after cell stimulation (Chen *et al*., 1992).

ERK1 and ERK2 are both activated through a cascade of kinases downstream of various receptor tyrosine kinases (RTKs) and cytokine receptors (Chang & Karin, 2001). In the MAPK pathway, ligand binding induces the activation of an RTK that initiates a signal transduction cascade, resulting in the activation of the GTPaseRas and the Ser/Thr kinase Raf. Activated Raf then binds to and phosphorylates MEK (MAPKK), resulting in its activation. MEK, a Ser/Thr and Tyr kinase, activates ERK by phosphorylating Thr202/Tyr204 and Thr185/Tyr187 of ERK1 and ERK2, respectively (Chang *et al*., 2003).

Activated ERK1 and ERK2 phosphorylate various substrates in multiple cellular compartments, including membrane proteins (CD120a, Syk, and calnexin), nuclear substrates (SRC-1, Pax6, NF-AT, Elk-1, MEF2, c-Fos, c-Myc, and STAT3), cytoskeletal proteins (neurofilaments and paxillin), and several MAPK-activated protein kinases (Chen *et al*., 2001).

Stress resistance in fibroblasts from Snell dwarf mice is accompanied by delays in kinetics of phosphorylation of ERK in the first hour after exposure to cadmium, paraquat, and H_2_O_2_, but not in cells exposed to UV light (Sun *et al*., 2009). The current work was undertaken to determine whether alterations in kinetics of ERK phosphorylation also distinguish fibroblasts of long-lived from short-lived species of mammals and birds.

## Results

**Figure 1:**
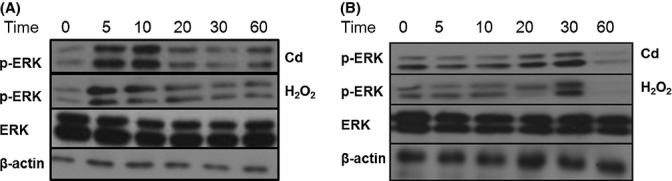
Representative autoradiographs of western blots for phosphorylated and total forms of ERK1/2 in skin-derived primary fibroblasts from rodents, in responses to cadmium (top row) or H_2_O_2_ (second row). Rows 3 and 4 show, respectively, total ERK and β-actin. Panel A: Red squirrel. Panel B: Chinchilla.

**Figure 2:**
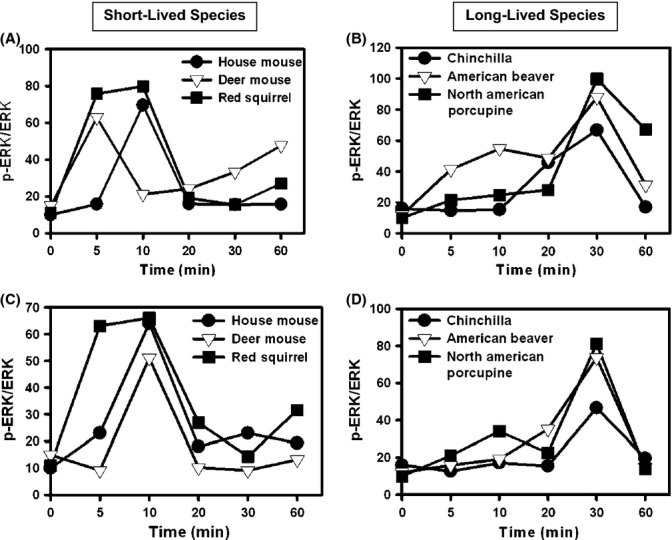
Phosphorylation of ERK1/2 in skin-derived primary fibroblasts from mammals represented as the ratio of phosphorylated ERK to total ERK at the indicated times after exposure to 80 μm cadmium (top panels) or 100 μm H_2_O_2_ (bottom panels). Panels A and C show three short-lived species, and panels B and D show three long-lived species.

To see whether interspecies differences in lifespan were associated with variation in the pace of stress-induced activation of the MEK/ERK system, we measured pERK levels in fibroblasts of 21 species of small mammals, including 18 species of rodents, with maximum lifespan (MLS) ranging from 2.2 to 23.4 years (Table S1, Supporting information). Cells were exposed to Cd or to H_2_O_2_ for 5–60 min, and the ratio of pERK/ERK measured as an index of MEK function. Figure 1 shows two representative images. For the red squirrel (left panel, maximum lifespan 9.8 years), ERK phosphorylation increases dramatically in the first 5 min after exposure to either stress and subsides to near-baseline (unstressed) levels by 30 min. For the chinchilla (right panel, maximum lifespan 17 years), ERK phosphorylation begins more gradually and continues to increase for 20–30 min before declining to baseline levels. Figure 2 shows the time course for the pERK/ERK ratio for six species in responses to cadmium (panels A and B) or to H_2_O_2_ (panels C and D). In cells from the relatively short-lived species (panels A and C), ERK phosphorylation increases quickly and then quickly returns to baseline. In cells from the longer-lived species (panels B and D), peak levels of pERK are not seen until the 30-min time point.

**Figure 3:**
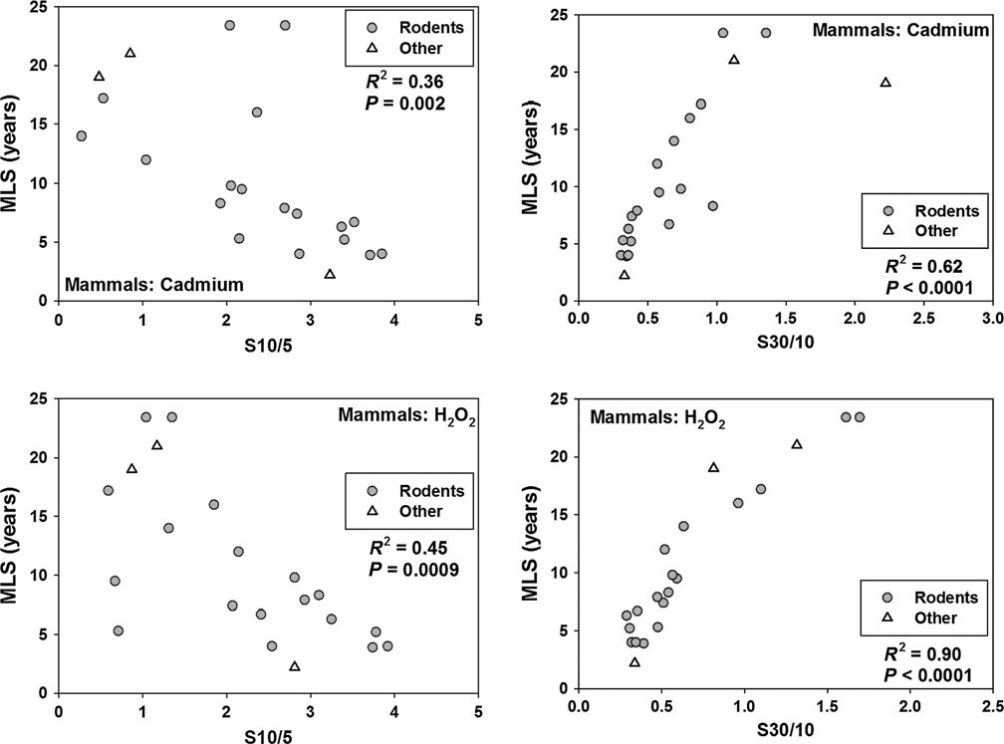
Scatterplots representing association of species lifespan (MLS in years) as a function of kinetic parameters (early slope S10/5 and later slope S30/10) for phosphorylation of ERK1/2 in skin-derived primary fibroblasts from 21 species of mammals. Upper panels show responses to cadmium, and lower panels show responses to H_2_O_2_. Rodent species indicated by circles and nonrodent species by triangles. The regression coefficients and p-values shown reflect calculations without adjustment for body mass.

For a more systematic analysis of the relation of lifespan to the kinetics of ERK phosphorylation, we calculated two indices for each species for each stress agent. We calculated S10/5 as the ratio of pERK/ERK at 10 min, divided by the pERK/ERK ratio at 5 min. High values of S10/5 indicate rapid induction of ERK phosphorylation. We calculated S30/10 as the ratio of pERK/ERK at 30 min divided by the ratio at 10 min. High values of S30/10 indicate species in which ERK phosphorylation remains high 30 min after stress exposure. We then used least squares regression to test the idea that S10/5 or S30/10 was associated with differences in MLS among the mammalian species. Figure 3 shows scatterplots of these associations, with the corresponding regression lines; significance levels for the regressions are provided in Table 1. Both for cadmium and for H_2_O_2_, longer lifespan is associated with low values of S10/5 (i.e. slower onset of ERK phosphorylation) and higher values of S30/10 (i.e. relatively prolonged maintenance of ERK phosphorylation). The associations are fairly strong, with *R*^2^ values ranging from 0.36 to 0.90, each significant at *P* < 0.002. When the analysis was limited to the 18 rodent species (Table S1, Supporting information), the association remained strong, with *P* < 0.03 for S10/5 in cadmium responses and *P* < 0.006 for the other three associations. When regression analyses were performed in which the species body mass and pERK kinetic indices were both included as predictor values, the association for the pERK indices remained significant, both for the mammalian data set as a whole and for the rodent species per se. Thus, we infer that skin-derived fibroblasts from longer-lived mammalian species, including rodent species, tend to activate MEK/ERK signals more slowly than cells from short-lived species and to retain elevated ERK activity for somewhat longer.

**Table 1:** Statistics from regression analyses for mammalian species

Endpoint	Dose (μm)	*P*-value	*R*^2^	*P*-value (log mass adjusted)	Rodents only *P*-value	Rodents only *P*-value (log mass adjusted)
S10/5, H_2_O_2_	300	0.001	0.45	0.003	0.006	0.02
S30/10, H_2_O_2_	300	0.000	0.90	0.000	0.000	0.000
S10/5, cadmium	140	0.002	0.36	0.001	0.03	0.02
S30/10, cadmium	140	0.000	0.62	0.000	0.000	0.001

Where *P*-value is given as 0.000, this represents *P* < 0.001. Other *P*-values are given rounded to the nearest significant figure.

**Figure 4:**
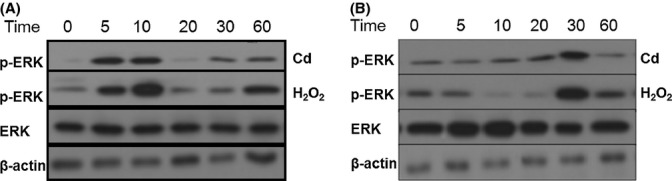
Representative autoradiographs of western blots for phosphorylated and total forms of ERK1/2 in skin-derived primary fibroblasts from rodents, in responses to cadmium (top row) or H_2_O_2_ (second row). Rows 3 and 4 show, respectively, total ERK and β-actin. Panel A: Carolina Wren. Panel B: Canada goose.

To see whether a similar relationship applied across a set of avian species, we also measured pERK levels in fibroblasts of 27 species of birds, with maximum lifespan (MLS) ranging from 7 to 50 years (Table S2, Supporting information). Similar to the assay used for mammals, avian cells were exposed to Cd or to H_2_O_2_ for 5–60 min, and the ratio of pERK/ERK was quantified as an index of MEK function. Figure 4 shows two representative images. For the Carolina Wren (left panel, maximum lifespan 9 years), ERK phosphorylation increases considerably in the first 5 min after exposure to either Cd or H_2_O_2_ and continues to increase through the 10-min time point, but then declines to near-baseline levels by 30 min. For the Canada goose (right panel, maximum lifespan 42 years), ERK phosphorylation increases more slowly, reaching a peak 30 min after exposure to either stress agent, before declining to baseline levels by 60 min.

**Figure 5:**
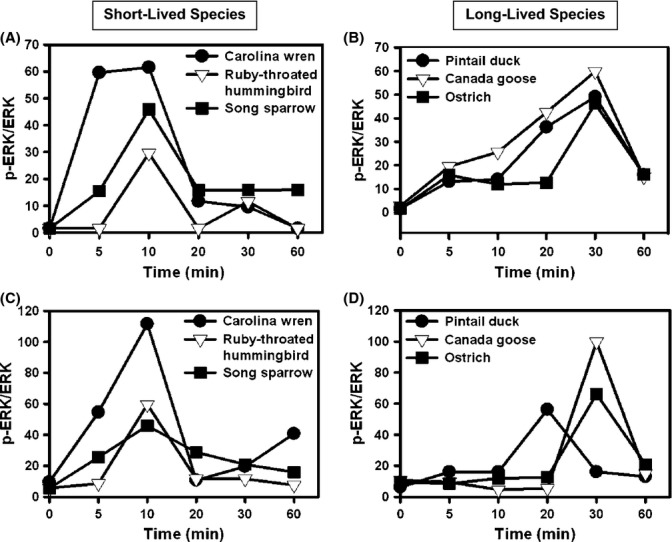
Phosphorylation of ERK1/2, in skin-derived primary fibroblasts from birds represented as the ratio of phosphorylated ERK to total ERK at the indicated times after exposure to 140 μm cadmium (top panels) or 300 μm H_2_O_2_ (bottom panels). Panels A and C show three short-lived species, and panels B and D show three long-lived species.

**Figure 6:**
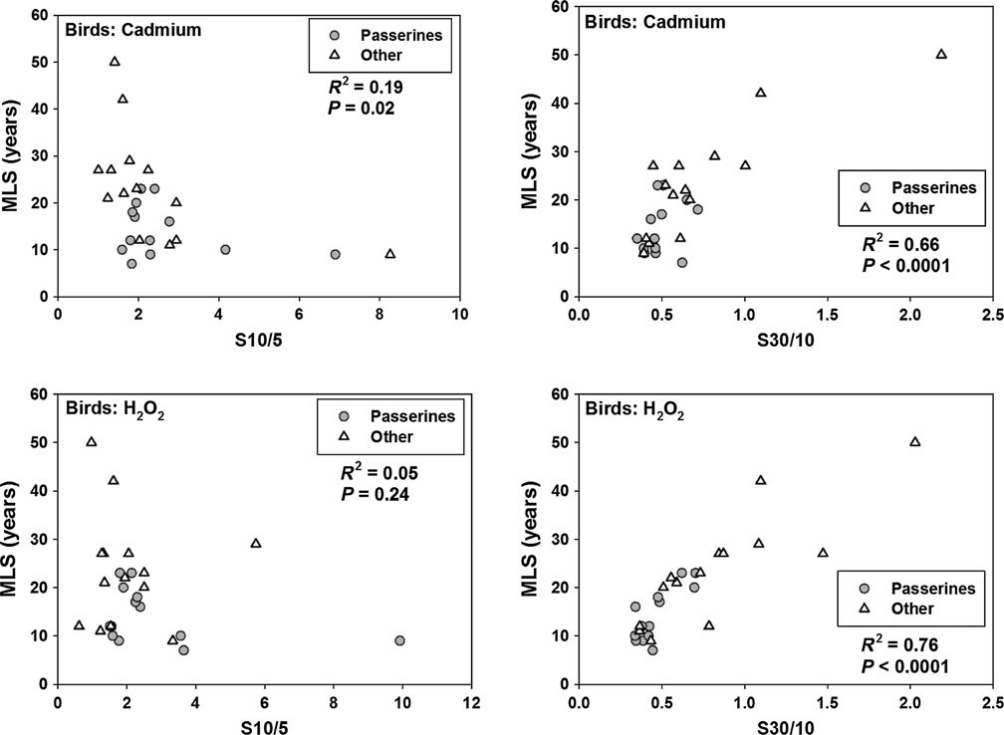
Scatterplots representing association of species lifespan (MLS in years) as a function of kinetic parameters (early slope S10/5 and later slope S30/10) for phosphorylation of ERK1/2 in skin-derived primary fibroblasts from 27 species of birds. Upper panels show responses to cadmium, and lower panels show responses to H_2_O_2_. Passerine species are indicated by circles, and nonpasserine species by triangles. The regression coefficients and p-values shown reflect calculations without adjustment for body mass.

Figure 5 shows quantitative estimates of the pERK/ERK ratio for six species in responses to cadmium (panels A and B) or to H_2_O_2_ (panels C and D). Panels A and C show short-lived species, and in these cases, pERK phosphorylation increases quickly in the early stages (time points 5 and 10 min) and then quickly returns to baseline (time points 20, 30, and 60 min). Panels B and D represent long-lived birds, and in these cases, peak levels of pERK do not appear until 20–30 min after stress exposure. Figure 6 shows the scatterplots and regression lines for all 27 avian species. Significance levels for the regressions are provided in Table 2. The associations are strongest for the S30/10 parameter, with *R*^2^ values of 0.66 and 0.76, each significant at *P* < 0.0001, in good agreement with the mammalian data set. For birds, there was less consistency in the early activation rate measured by the S10/5 parameter: The association was significant for cadmium (*R*^2^ = 0.16, *P* = 0.02) but not for H_2_O_2_ (*R*^2^ = 0.02, *P* = 0.25). A regression analysis in which both log (mass) and the pERK/ERK indices were used as predictors of maximum lifespan, also shown in Table 2, reduced the strength of the association, and only S30/10, for H_2_O_2_, remained at *P* < 0.05 in these regressions.

**Table 2:** Statistics from regression analyses for avian species

Endpoint	Dose (μm)	*P*-value	*R*^2^	*P*-value (log mass adjusted)
S10/5, H_2_O_2_	300	0.24	0.05	0.6
S30/10, H_2_O_2_	300	0.000	0.76	0.02
S10/5, cadmium	140	0.02	0.19	0.9
S30/10, cadmium	140	0.000	0.66	0.12

**Figure 7:**
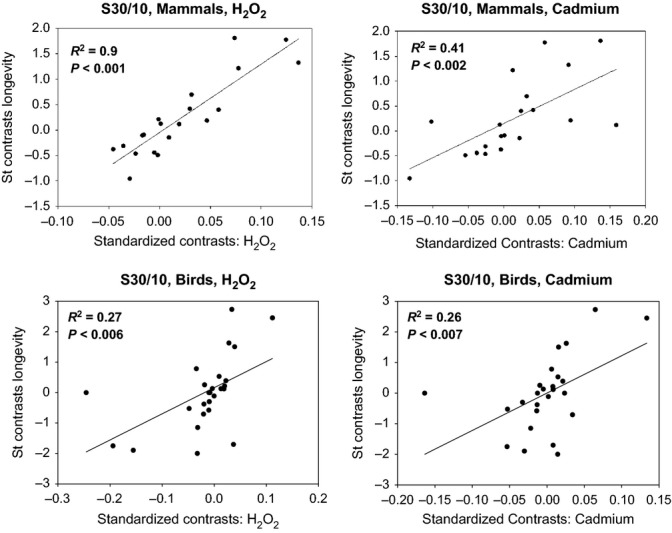
Association of lifespan to S30/10 ERK responses with standardized contrast adjustments for phylogenetic relationships. Mammals are shown in the top panels and bird species in the bottom panels, with H_2_O_2_ data on the left and cadmium data on the right.

Standard regression methods make the assumption that the data for each species, both lifespan and ERK kinetic data, are independent, but this assumption is not correct, because closely related species reflect fewer evolutionary changes than species further from their most recent shared ancestor (Felsenstein, 1985). We therefore evaluated our data using the standardized independent contrast method (Garland & Adolph, 1994), which weights associations between lifespan and ERK based on the phylogenetic distances among groups of species. For mammals, we found no significant relationship for the S10/5 (early response) parameter, either for H_2_O_2_ (*r*^2^ = 0.13, *P* = 0.12) or for cadmium (*r*^2^ = 0.09, *P* = 0.2), but the relationship for the S30/10 parameter remained strong and statistically significant for both sources of stress (Fig. 7, top panels). Similarly, S10/5 was not significantly related to species lifespan among bird species (*r*^2^ = 0.0004, *P* = 0.93 for H_2_O_2_ and *r*^2^ = 0.1, *P* = 0.13 for cadmium), but S30/10 showed a significant relationship for both sources of stress (Fig. 7, bottom panels). Because passerine species make up nearly half of the bird species in our collection, we repeated the independent contrast analysis using an approach that collapsed all of the passerine species to a single point. Despite the fact that this analysis reduced our functional sample size by nearly half, there was still a suggestion of association for S10/5 for the cadmium data set (*r*^2^ = 0.27, *P* = 0.055) but not for the H_2_O_2_ stress (*r*^2^ = 0.01, *P* = 0.7), and the association with S30/10 levels remained statistically significant (cadmium: *r*^2^ = 0.35, *P* = 0.03; H_2_O_2_: *r*^2^ = 0.31, *P* = 0.04, data not shown). We conclude that the relationship between sustained ERK activation (S30/10) and lifespan in mammals and birds is not an artifact of shared phylogenetic descent, but we cannot rule out this idea for the early stages of ERK activation (S10/5) using our current data set.

## Discussion

Primary skin fibroblast cell lines isolated from adults of 27 avian species (seven orders, with maximum lifespan ranging from 7 to 50 years) and 21 mammalian species (mostly rodents, maximum lifespan range from 2 to 23 years) were used to examine the relationship between species lifespan and the pace of response to cadmium and H_2_O_2_ as measured by changes in phosphorylation of the stress-activated protein kinases ERK1/2. Previous work from our laboratory has shown that primary fibroblast cultures established from the skin of long-lived mutant Snell dwarf, Ames dwarf, and growth hormone receptor knockout (GHRKO) mice are resistant to lethal injury induced by cadmium and H_2_O_2_ (Murakami *et al*., 2003; Salmon *et al*., 2005). Studies of the Snell and GHRKO fibroblasts showed slower rates of phosphorylation of ERK1/2 after exposure to peroxide, cadmium, or paraquat (Sun *et al*., 2009). Our current results suggest that differences in the kinetics of stress-induced activation of ERKs are also seen in cross-species contrasts. For both birds and mammals, species with longer lifespan show slower onset of ERK phosphorylation, in that the ratio of pERK at 30 min to pERK at 10 min is higher in long-lived species, both for cadmium and for H_2_O_2_ exposure. For the mammals, the same pattern can be seen in the first 10 min after stress exposure, with high ratios of pERK at 10 min to pERK at 5 min, characteristic of shorter-lived species for both sources of cellular injury. The regression coefficients for rodents, which made up 18 of the 21 tested mammals, are similar to those seen in the larger mammalian data set. In qualitative terms, cells from shorter-lived species tend to turn on ERK activity sooner, and turn it off sooner, than cells from the longer-lived species.

Interpretation of regression analyses after adjustment for species differences in body mass is complicated by several considerations. Because larger species also tend to live longer than smaller ones, traits that are closely related to mass, such as metabolic rate, would show a correlation with longevity even if there were no causal links between the trait and the rate of aging. For this reason, some analysts (Speakman, 2005) place little value on associations of longevity with physiological measurements across species unless these associations survive correction for species mass. Other workers (Hulbert, 2005; Hulbert *et al*., 2006) note that adjustment for mass is likely to obscure associations that do indeed reflect causal associations and that diluting statistical trends by adjustment for a common covariate (mass) will increase type II errors. We have therefore reported the strength of association for our ERK parameters both with and without adjustment for species mass (Tables 1 and 2), and note that the significant associations seen for both kinetic parameters, for both stresses, survive adjustment for species mass in the rodents, but are weakened by this adjustment (*P* = 0.02 for H_2_O_2_ and *P* = 0.12 for cadmium) among the bird species.

Both our mammal and bird data are heavily weighted toward certain phylogenetic groups. Eighteen of our 21 mammal species are rodents, and 13 of our 27 bird species are passerines. However, even when we analyzed the data adjusting for phylogeny, the pattern for prolonged phosphorylation of ERK in our longer-lived species in response to both H_2_O_2_ and cadmium persisted.

There is a good deal of evidence supporting the idea that differences in response to cellular stresses may contribute to variation in the rate of aging and in lifespan (Johnson *et al*., 1996). Enhanced stress resistance is associated with extended longevity in worms, flies, and yeast, with a significantly positive relationship between individual ability to respond to stress and its longevity (Johnson *et al*., 1996). Among isogenic worms, those individuals with a high capacity to react to thermal stress are relatively long-lived. Studies of fibroblasts have also shown relationships between species lifespan and resistance to lethal stress in mammals (Kapahi *et al*., 1999), birds (Harper *et al*., 2011), and rodents (Harper *et al*., 2007), as well as differences in clonal growth patterns and responses to contact inhibition (Seluanov *et al*., 2008). Our data extend these earlier reports by suggesting altered kinetics of ERK activation as indices of upstream responses to stress and potentially as mediators of cellular responses to injury.

Even though it is known that ERK1/2 is activated by oxidative injury, the exact mechanism of activation of MEK and ERK by agents such as cadmium and hydrogen peroxide is not yet well defined. The pathways by which these agents induce MEK and ERK activity may well differ. For instance, H_2_O_2_ has been proven to directly stimulate ERK-1 and ERK-2 phosphorylation in human fibroblasts after 5 min of exposure to H_2_O_2_, with the activity peaking after 20 min (Ceolotto *et al*., 2004), via a sequential activation of PKC, Raf-1, and MEK1 (Abe *et al*., 1998). Cadmium also causes oxidative injury as a secondary effect of its inhibition of a multitude of enzymatic reactions, but does not seem to interfere directly with cellular oxygen metabolism (Jonak *et al*., 2004).

We do not know the molecular basis for the interspecies differences in the pace of ERK activation in our cell lines. The Ras/Raf/MEK/ERK cascade couples signals from cell surface receptor tyrosine kinases (RTKs) to transcription factors, which in turn regulate gene expression (Kolch, 2005). In some cell systems, activation of ERK1/2 promotes cell survival, while activation of JNK or p38 induces apoptosis (Marshall, 1995). In other cell systems, activation of ERKs can generate pro-apoptotic signals as well (Chandra *et al*., 2002; Recchia *et al*., 2004).

ERK activation by 17β-estradiol differs between osteoblasts and osteoclasts; in the former cell type, the effect of the hormone was brief, with return to baseline within 30 min, and protected against apoptosis, while the response of the latter cell type was sustained for at least 24 h and promoted cell death, with corresponding differences in the timing of nuclear accumulation of ERK (Chen *et al*., 2005). The timing and duration of nuclear accumulation of ERKs are modulated by nuclear phosphatases responsible for ERK dephosphorylation including dual-specificity phosphatases (DUSPs) and MAPK-selective phosphatases (MKP) (Khokhlatchev *et al*., 1998; Woods & Johnson, 2006). More generally, transient modulation of MAPK (and specifically ERK) activity is thought to reflect complex interactions involving effects of spatially restricted phosphatases and scaffolding proteins, including negative feedback to limit continued stimulation through upstream kinases (Kholodenko & Birtwistle, 2009). Our data, limited to the first 60 min after stress exposure, do not reveal whether additional oscillations in ERK function might be detected over a longer time course and do not provide insights into species-specific variation in ERK-binding scaffolds or spatial localization of key kinases and phosphatases. Computational models (Bhalla *et al*., 2002) of growth factor action suggest that variations in a MAPK-specific phosphatase, MKP, can control whether extracellular signals lead to graded ERK responses or alternately to an all-or-none switch between two stable states. Studies of 3T3 cells are consistent with the idea that arachidonic acid derivatives can also provide negative feedback signals on ERK activity by the control of protein kinase C function (Bhalla *et al*., 2002). Clarification of the potential role of feedback signals in species-specific modulation of ERK kinase, and cellular resistance to lethal stress, will provide attractive topics for additional investigation.

The outcome of c-Src inhibition of osteoclasts is also regulated by the timing of pERK activation (Recchia *et al*., 2004). In this system, c-Src inhibition led to rapid loss of pERK within 15 min, followed by the increase in pERK, 2 h later, to levels above those seen in untreated control cells. Pharmacologic inhibition of this secondary increase in pERK reduced apoptosis. In a third system, activation of c-Fos was shown to require only transient pERK activity in CCL9 hamster fibroblasts, whereas sustained pERK was needed for the activation of JunB and Fra-1 and for cell cycle entry (Balmanno & Cook, 1999).

Similarly, sustained phosphorylation and nuclear localization of ERK regulate S phase entry in Swiss 3T3 fibroblasts cells. Both PDGF and EGF lead, within 10–15, min to phosphorylated active ERK and translocation of pERK to the nucleus. ERK phosphorylation is transient after EGF exposure, returning to baseline within 30–45 min, while PDGF-induced pERK is sustained for at least 240 min, leading to S phase entry (Murphy *et al*., 2002).

Each of these experimental systems suggests that modulation of the kinetics of ERK activity and deactivation can have functional consequences, but further work will be needed to evaluate the causes and consequences of differential kinetics of ERK activation in fibroblasts from long-lived and short-lived species. One possibility is that c-Fos may function as a molecular sensor regulating consequences downstream of ERK phosphorylation and nuclear localization (Murphy *et al*., 2002). Newly synthesized c-Fos protein has a half-life of about 30–45 min (Curran *et al*., 1984), which can be extended to 2 h or more after ERK-mediated phosphorylation (Chen *et al*., 1996). Therefore, in cells where ERK is rapidly inactivated, c-Fos is present in the nucleus, but is not phosphorylated, and is therefore unstable and degraded. In contrast, delayed inactivation of ERK can result in an efficient phosphorylation of c-Fos and its stabilization for several hours (Marshall, 1995; New *et al*., 2001; Murphy *et al*., 2002). Alteration in the timing of ERK-dependent signals may contribute to species-specific variation in cellular properties *in vitro* and give clues to cellular properties by which evolution controls aging rate and disease risk across species.

## Experimental procedures

### Cell culture and conditions

Fibroblast cultures from 27 bird species and 21 mammalian species were obtained from cells grown from cryopreserved aliquots (P1) stored in liquid N_2_ at 10^6^ cells mL^−1^ in DMEM supplemented with 40% fetal bovine serum and dimethyl sulfoxide (DMSO) at a final concentration of 20%. The establishment of the cell lines used in this research has been described previously (Harper *et al*., 2007, 2011). Cells were grown at 37°C in a humidified incubator with 5% CO_2_ in 3% O_2_, as there is evidence that exposure to 21% O_2_ can lead to early growth crisis relative to cells grown at lower oxygen tension (Busuttil *et al*., 2003; Maynard & Miller, 2006). Each species was represented by a single donor in this study.

Cells thawed from cryopreserved aliquots were cultured for 7–14 days in Dulbecco’s modified Eagle’s medium (DMEM) [high glucose variant (4.5 mg mL^−1^), with sodium pyruvate (110 mg mL^−1^) containing 10 mm HEPES], supplemented with 10% heat-inactivated fetal bovine serum, 100 units mL^−1^ penicillin, and 100 μg mL^−1^ streptomycin, and 2% heat-inactivated chicken serum was added for bird cell lines. These cells were then harvested and used for the assessment of ERK activation kinetics.

### Reagents and antibodies

Cadmium chloride hemi (pentahydrate) (CdCl_2_·2_1/2_H_2_O) and dimethyl sulfoxide (DMSO) were purchased from Sigma-Aldrich (St Louis, MO, USA). CdCl_2_ was dissolved in H_2_O, with the pH of 5 μm CdCl_2_ at 7.1. FBS was from Invitrogen/GIBCO (Grand Island, NY, USA). Antibodies against phospho-p44/42 ERK (Thr202/Tyr204) and p44/42 ERK were from Cell Signaling Technology (Beverly, MA, USA); β-actin was from Sigma-Aldrich Corp. (St. Louis, MO, USA). Goat anti-rabbit and goat anti-mouse antibodies were from Santa Cruz Biotechnology, Inc. (Santa Cruz, CA, USA).

### Cd and H_2_O_2_ dosage relative to LD50

Because bird fibroblasts are more resistant to lethal stress than rodent fibroblasts (Harper *et al*., 2011), we used different concentrations of cadmium and H_2_O_2_ for the induction of ERK phosphorylation, depending on the clade. Mammalian cells were exposed to 80 μm cadmium and 100 μm peroxide, in each case corresponding roughly to twice the mean LD50 for rodent species in our previous publication (Harper *et al*., 2011). For bird cells, we used 140 μm cadmium and 300 μm peroxide, again based on previous estimates of LD50.

### Cellular stress assay

Cells were placed at 5 × 10^5^ in each well of a six-well plate and incubated overnight in 2 mL of complete DMEM in 3% O_2_. On the next day, the medium was replaced with DMEM containing 2% BSA but without serum. The following day, the cells were washed in PBS and placed on ice. Cadmium or peroxide dissolved in DMEM was added, and the culture dish placed at 37° for intervals of 5–60 min, after which cells were washed once in PBS and harvested for immunoblot analysis.

### Western blot analysis

Cells were washed with ice-cold phosphate-buffered saline (PBS) (140 mm NaCl, 3 mm KCl, 6 mm Na_2_HPO_4_, and 1 mm KH_2_PO_4_, pH 7.4) and then harvested in 100 μL of lysis buffer [20-mm Tris (pH 7.5), 150 mm NaCl, 1% Triton 100], with protease inhibitor cocktail and phosphatase inhibitor cocktails (Sigma-Aldrich Corp.). Lysates were centrifuged at 15300 *g* for 30 min at 4°C, and supernatants collected. Protein concentration was measured using the bicinchoninic acid assay (Pierce Corp., Rockford, IL, USA). The protein samples were mixed with 2× loading buffer (Bio-Rad Laboratories, Inc., Hercules, CA, USA) and heated at 100°C for 5 min. Ten micrograms of total protein was then subjected to SDS polyacrylamide gel electrophoresis and separated according to size using Criterion XT Precast Gel (Bio-Rad Laboratories) for 60 min at 150 V. The resolved proteins were then wet-transferred to nitrocellulose membranes for 1 h at 100 V and blocked with 5% BSA in Tris buffer saline (TBS, pH 7.6) containing 0.05% Tween 20. Proteins were then wet-transferred for 1 h at 100 V at room temperature. The membranes were then washed with TBST and incubated with the primary antibody diluted in the appropriate blocking solution at 4°C overnight with shaking. After incubation, the protein blots were washed five times (15 min each) with TBST and incubated with the appropriate secondary antibody. The protein blots were visualized by the incubation with enhanced chemiluminescence reagent from Pierce (Thermo Scientific, Rockford, IL, USA); the luminescent signals were analyzed using an ImageQuant LAS 4000 scanner from GE Healthcare (Piscataway, NJ, USA). Quantification of immunoblot signals was performed using the ImageQuant software package (Molecular Dynamics, Sunnyvale, CA, USA).

### Statistical analysis

Statistical analysis was carried out using Logger Pro, using linear regression between the reported maximum lifespan and the level of phosphorylation of ERK for each of the 27 species for birds and the 21 mammal species. Information on adult body mass and maximum recorded lifespan was obtained from the Age Database of Animal Ageing and Longevity, at http://genomics.senescence.info/species/. Phylogenetic analyses were performed using the method of standardized independent contrasts (Felsenstein, 1985; Garland & Adolph, 1994). Mammalian phylogenies were constructed from the studies of Mercer & Roth (2003), Fabre *et al*. (2012), and O’Leary *et al*. (2013). Avian phylogenies were from the study of Jetz *et al*. (2012).

## Supporting Information

**Fig. S1** Phylogenetic relationships among mammalian species. **Fig. S2** Phylogenetic relationships among avian species. **Table S1** Taxonomy of birds used in this study, with maximum life span and mass. **Table S2** Taxonomy of mammals used in this study, with maximum life span and mass.

## References

[b1] Abe MK, Kartha S, Karpova AY, Li J, Liu PT, Kuo W-L, Hershenson MB (1998). Hydrogen peroxide activates extracellular signal-regulated kinase via protein kinase C, Raf-1, and MEK1. Am. J. Respir. Cell Mol. Biol.

[b2] Austad SN (2009). Comparative biology of aging. J. Gerontol. A Biol. Sci. Med. Sci.

[b3] Balmanno K, Cook S (1999). Sustained MAP kinase activation is required for the expression of cyclin D1, p21Cip1 and a subset of AP-1 proteins in CCL39 cells. Oncogene.

[b4] Bhalla US, Ram PT, Iyengar R (2002). MAP kinase phosphatase as a locus of flexibility in a mitogen-activated protein kinase signaling network. Science.

[b5] Brown-Borg HM, Borg KE, Meliska CJ, Bartke A (1996). Dwarf mice and the ageing process. Nature.

[b6] Busuttil RA, Rubio M, DollA C, Campisi J, Vijg J (2003). Oxygen accelerates the accumulation of mutations during the senescence and immortalization of murine cells in culture. Aging Cell.

[b7] Ceolotto G, Bevilacqua M, Papparella I, Baritono E, Franco L, Corvaja C, Mazzoni M, Semplicini A, Avogaro A (2004). Insulin generates free radicals by an NAD(P)H, phosphatidylinositol 3′-kinase-dependent mechanism in human skin fibroblasts ex vivo. Diabetes.

[b8] Chandra D, Liu J, Tang DG (2002). Early mitochondrial activation and cytochrome c up-regulation during apoptosis. J. Biol. Chem.

[b9] Chang L, Karin M (2001). Mammalian MAP kinase signalling cascades. Nature.

[b10] Chang F, Steelman L, Lee JT, Shelton JG, Navolanic PM, Blalock WL, Franklin RA, McCubrey JA (2003). Signal transduction mediated by the Ras/Raf/MEK/ERK pathway from cytokine receptors to transcription factors: potential targeting for therapeutic intervention. Leukemia.

[b11] Chen RH, Sarnecki C, Blenis J (1992). Nuclear localization and regulation of erk- and rsk-encoded protein kinases. Mol. Cell. Biol.

[b12] Chen RH, Juo P, Curran T, Blenis J (1996). Phosphorylation of c-Fos at the C-terminus enhances its transforming activity. Oncogene.

[b13] Chen Z, Gibson T, Robinson F, Silvestro L, Pearson G, Xu B, Wright A, Vanderbilt C, Cobb MH (2001). MAP kinases. Chem. Rev.

[b14] Chen JR, Plotkin L, Aguirre JI, Han L, Jilka RL, Kousteni S, Bellido T, Manolagas SC (2005). Transient versus sustained phosphorylation and nuclear accumulation of ERKs underlie anti-versus pro-apoptotic effects of estrogens. J. Biol. Chem.

[b15] Costa M, Marchi M, Cardarelli F, Roy A, Beltram F, Maffei L, Ratto GH (2006). Dynamic regulation of ERK2 nuclear translocation and mobility in living cells. J. Cell Sci.

[b16] Curran T, Miller A, Zokas L, Verma IM (1984). Viral and cellular fos proteins: a comparative analysis. Cell.

[b17] Dammann P, Sumbera R, Massmann C, Scherag A, Burda H (2011). Extended longevity of reproductives appears to be common in Fukomys mole-rats (Rodentia, Bathyergidae). PLoS ONE.

[b18] Fabre PH, Hautier L, Dimitrov D, Douzery EJ (2012). A glimpse on the pattern of rodent diversification: a phylogenetic approach. BMC Evol. Biol.

[b19] Felsenstein J (1985). Phylogenies and the comparative method. Am. Nat.

[b20] Flurkey K, Papaconstantinou J, Miller RA, Harrison DE (2001). Lifespan extension and delayed immune and collagen aging in mutant mice with defects in growth hormone production. Proc. Natl Acad. Sci. USA.

[b21] Garland T, Adolph SC (1994). Why Not to Do 2-Species Comparative-Studies - Limitations on Inferring Adaptation. Physiol. Zool.

[b22] Harper JM, Salmon AB, Leiser SF, Galecki AT, Miller RA (2007). Skin-derived fibroblasts from long-lived species are resistant to some, but not all, lethal stresses and to the mitochondrial inhibitor rotenone. Aging Cell.

[b23] Harper JM, Wang M, Galecki AT, Ro J, Williams JB, Miller RA (2011). Fibroblasts from long-lived bird species are resistant to multiple forms of stress. J. Exp. Biol.

[b24] Harrison DE, Strong R, Sharp ZD, Nelson JF, Astle CM, Flurkey K, Nadon NL, Wilkinson JE, Frenkel K, Carter CS, Pahor M, Javors MA, Fernandez E, Miller RA (2009). Rapamycin fed late in life extends lifespan in genetically heterogeneous mice. Nature.

[b25] Holmes DJ, Fluckiger R, Austad SN (2001). Comparative biology of aging in birds: an update. Exp. Gerontol.

[b26] Hulbert AJ (2005). On the importance of fatty acid composition of membranes for aging. J. Theor. Biol.

[b27] Hulbert AJ, Faulks SC, Buffenstein R (2006). Oxidation-resistant membrane phospholipids can explain longevity differences among the longest-living rodents and similarly-sized mice. J. Gerontol. A Biol. Sci. Med. Sci.

[b28] Jetz W, Thomas GH, Joy JB, Hartmann K, Mooers AO (2012). The global diversity of birds in space and time. Nature.

[b29] Johnson TE, Lithgow GJ, Murakami S (1996). Hypothesis: interventions that increase the response to stress offer the potential for effective life prolongation and increased health. J. Gerontol. A Biol. Sci. Med. Sci.

[b30] Jonak C, Nakagami H, Hirt H (2004). Heavy metal stress. Activation of distinct mitogen-activated protein kinase pathways by copper and cadmium. Plant Physiol.

[b31] Kapahi P, Boulton ME, Kirkwood TB (1999). Positive correlation between mammalian life span and cellular resistance to stress. Free Radical Biol. Med.

[b32] Khokhlatchev AV, Canagarajah B, Wilsbacher J, Robinson M, Atkinson M, Goldsmith E, Cobb MH (1998). Phosphorylation of the MAP kinase ERK2 promotes its homodimerization and nuclear translocation. Cell.

[b33] Kholodenko BN, Birtwistle MR (2009). Four-dimensional dynamics of MAPK information processing systems. Wiley interdisciplinary reviews. Syst. Biol. Med.

[b34] Kolch W (2005). Coordinating ERK/MAPK signalling through scaffolds and inhibitors. Nat. Rev. Mol. Cell Biol.

[b35] Leiser SF, Miller RA (2009). Nrf2 Signaling: a mechanism for cellular stress resistance in long-lived mice. Mol. Cell. Biol.

[b36] Marshall CJ (1995). Specificity of receptor tyrosine kinase signaling: transient versus sustained extracellular signal-regulated kinase activation. Cell.

[b37] Maynard SP, Miller RA (2006). Fibroblasts from long-lived Snell dwarf mice are resistant to oxygen-induced *in vitro* growth arrest. Aging Cell.

[b38] Mercer JM, Roth VL (2003). The effects of Cenozoic global change on squirrel phylogeny. Science.

[b39] Murakami S, Salmon A, Miller RA (2003). Multiplex stress resistance in cells from long-lived dwarf mice. FASEB J.

[b40] Murphy LO, Smith S, Chen RH, Fingar DC, Blenis J (2002). Molecular interpretation of ERK signal duration by immediate early gene products. Nat. Cell Biol.

[b41] Narasimhan P, Liu J, Song YS, Massengale JL, Chan PH (2009). VEGF Stimulates the ERK 1/2 signaling pathway and apoptosis in cerebral endothelial cells after ischemic conditions. Stroke.

[b42] New L, Li Y, Ge B, Zhong H, Mansbridge J, Liu K, Han J (2001). SB203580 promotes EGF-stimulated early morphological differentiation in PC12 cell through activating ERK pathway. J. Cell. Biochem.

[b43] O’Leary MA, Bloch JI, Flynn JJ, Gaudin TJ, Giallombardo A, Giannini NP, Goldberg SL, Kraatz BP, Luo ZX, Meng J, Ni X, Novacek MJ, Perini FA, Randall ZS, Rougier GW, Sargis EJ, Silcox MT, Simmons NB, Spaulding M, Velazco PM, Weksler M, Wible JR, Cirranello AL (2013). The placental mammal ancestor and the post-K-Pg radiation of placentals. Science.

[b44] Orentreich N, Matias JR, DeFelice A, Zimmerman JA (1993). Low methionine ingestion by rats extends life span. J. Nutr.

[b45] Parrinello S, Samper E, Krtolica A, Goldstein J, Melov S, Campisi J (2003). Oxygen sensitivity severely limits the replicative lifespan of murine fibroblasts. Nat. Cell Biol.

[b46] Recchia I, Rucci N, Funari A, Migliaccio S, Taranta A, Longo M, Kneissel M, Susa M, Fabbro D, Teti A (2004). Reduction of c-Src activity by substituted 5,7-diphenyl-pyrrolo[2,3-d]-pyrimidines induces osteoclast apoptosis *in vivo* and *in vitro*. Involvement of ERK1/2 pathway. Bone.

[b47] Roux PP, Blenis J (2004). ERK and p38 MAPK-activated protein kinases: a family of protein kinases with diverse biological functions. Microbiol. Mol. Biol. Rev.

[b48] Salmon AB, Murakami S, Bartke A, Kopchick J, Yasumura K, Miller RA (2005). Fibroblast cell lines from young adult mice of long-lived mutant strains are resistant to multiple forms of stress. Am. J. Physiol. Endocrinol. Metab.

[b49] Seluanov A, Hine C, Bozzella M, Hall A, Sasahara TH, Ribeiro AA, Catania KC, Presgraves DC, Gorbunova V (2008). Distinct tumor suppressor mechanisms evolve in rodent species that differ in size and lifespan. Aging Cell.

[b50] Speakman JR (2005). Correlations between physiology and lifespan–two widely ignored problems with comparative studies. Aging Cell.

[b51] Sun LY, Steinbaugh MJ, Masternak MM, Bartke A, Miller RA (2009). Fibroblasts from long-lived mutant mice show diminished ERK1/2 phosphorylation but exaggerated induction of immediate early genes. Free Radical Biol. Med.

[b52] Weindruch R, Sohal RS (1997). Seminars in medicine of the Beth Israel Deaconess Medical Center. Caloric intake and aging. N. Engl. J. Med.

[b53] Woods DC, Johnson A (2006). Phosphatase activation by epidermal growth factor family ligands regulates extracellular regulated kinase signaling in undifferentiated hen granulosa cells. Endocrinology.

