# Supplementary material for: Fibroblasts from long-lived species of mammals and birds show delayed, but prolonged, phosphorylation of ERK
Source: Aging Cell. 2013 Nov 13;13(2):283–91. doi: 10.1111/acel.12172 (PMC3954945; doi:10.1111/acel.12172)

**Supplementary tables:**

Supplementary Table 1: Taxonomy of birds used in this study, with maximum life span and mass.

| **Common Name** | **Latin Name** | **Order** | **Family** | **MLS(yrs)** | **Mass (g)** | **Cad LD50** | **Cad S10_5** | **CadS30_10** | **Perox LD50** | **PeroxS10_5** | **PeroxS30_10** |
| --- | --- | --- | --- | --- | --- | --- | --- | --- | --- | --- | --- |
| Yellow-rumped Warbler | Dendroicacoronata | Passeriformes | Parulidae | 7 | 13 | 252.42 | 1.83 | 0.62 | 252.42 | 3.65 | 0.44 |
| Carolina Wren | Thryothorusludovicianus | Passeriformes | Troglodytidae | 9 | 18 | 45.58 | 6.90 | 0.46 | 45.58 | 9.92 | 0.39 |
| House wren | Troglodytes aedon | Passeriformes | Troglodytidae | 9 | 10 | 147.16 | 2.30 | 0.39 | 147.16 | 1.77 | 0.34 |
| Ruby-throated Hummingbird | Archilochuscolubris | Apidoformes | Trochilidae | 9 | 3 | 23.67 | 8.26 | 0.39 | 23.67 | 3.33 | 0.43 |
| Red-eyed Vireo | Vireo olivaceus | Passeriformes | Vireonidae | 10 | 17 | 61.58 | 4.17 | 0.46 | 61.58 | 3.56 | 0.42 |
| White-breasted Nuthatch | Sittacarolinensis | Passeriformes | Sittidae | 10 | 21 | 14.71 | 1.59 | 0.39 | 14.71 | 1.59 | 0.34 |
| Killdeer | Charadriusvociferus | Charadriiformes | Charadriidae | 11 | 88 | 88.59 | 2.78 | 0.42 | 88.59 | 1.23 | 0.36 |
| Spotted Sandpiper | Actitismacularius | Charadriiformes | Scolopacidae | 11 | 23 | 85.30 | 2.94 | 0.40 | 85.30 | 1.53 | 0.37 |
| Chipping Sparrow | Spizellapasserina | Passeriformes | Emberizidae | 12 | 12 | 64.73 | 2.29 | 0.35 | 64.73 | 1.55 | 0.42 |
| Downy Woodpecker | Picoidespubescens | Piciformes | Picidae | 12 | 26 | 54.87 | 2.03 | 0.61 | 54.87 | 0.61 | 0.78 |
| Song Sparrow | Melospizamelodia | Passeriformes | Emberizidae | 12 | 34 | 52.51 | 1.80 | 0.46 | 52.51 | 1.51 | 0.38 |
| Barn Swallow | Hirundorustica | Passeriformes | Hirundinidae | 16 | 18 | 117.50 | 2.77 | 0.43 | 117.50 | 2.39 | 0.34 |
| American Robin | Turdusmigratorius | Passeriformes | Turdidae | 17 | 76 | 77.79 | 1.91 | 0.50 | 77.79 | 2.25 | 0.49 |
| Gray Catbird | Dumetellacarolinensis | Passeriformes | Mimidae | 18 | 35 | 75.91 | 1.85 | 0.72 | 75.91 | 2.30 | 0.48 |
| Northern Shoveler | Anasclypeata | Anseriformes | Anatidae | 20 | 613 | 33.08 | 2.94 | 0.67 | 33.08 | 2.50 | 0.51 |
| Crow | Corvusbrachyrhynchos | Passeriformes | Corvidae | 20 | 385 | - | 1.94 | 0.65 | - | 1.90 | 0.70 |
| Red-bellied Woodpecker | Melanerpescarolinus | Piciformes | Picidae | 21 | 73 | - | 1.24 | 0.57 | - | 1.35 | 0.59 |
| Gadwall | Anasstrepera | Anseriformes | Anatidae | 22 | 791 | 62.67 | 1.63 | 0.64 | 62.67 | 1.94 | 0.56 |
| Common Grackle | Quiscalusquiscula | Passeriformes | Icteridae | 23 | 111 | 96.12 | 2.40 | 0.51 | 96.12 | 1.80 | 0.70 |
| Starling | Sturnus vulgaris | Passeriformes | Sturnidae | 23 | 74 | 40.09 | 2.06 | 0.47 | 40.09 | 2.14 | 0.62 |
| Wood Duck | Aix sponsa | Anseriformes | Anatidae | 23 | 453 | 76.15 | 1.95 | 0.52 | 76.15 | 2.50 | 0.73 |
| Pheasant | Phasianuscolchicus | Galliformes | Phasianidae | 27 | 1095 | 35.67 | 2.24 | 0.45 | 35.67 | 1.32 | 0.84 |
| Green-winged Teal | Anascrecca | Anseriformes | Anatidae | 27 | 344 | 36.70 | 1.32 | 0.60 | 36.70 | 2.05 | 0.87 |
| Pintail Duck | Anasacuta | Anseriformes | Anatidae | 27 | 721 | 38.85 | 1.00 | 1.00 | 38.85 | 1.27 | 1.47 |
| Mallard | Anasplatyrhynchos | Anseriformes | Anatidae | 29 | 1048 | 56.28 | 1.78 | 0.82 | 56.28 | 5.74 | 1.08 |
| Canada Goose | Brantacanadensis | Anseriformes | Anatidae | 42 | 3200 | 311.27 | 1.61 | 1.10 | 311.27 | 1.61 | 1.10 |
| Ostrich | Struthiocamelus | Struthioniformes | Struthionidae | 50 | 111000 | 396.74 | 1.40 | 2.19 | 396.74 | 0.97 | 2.03 |

Supplementary Table 2: Taxonomy of mammals used in this study, with maximum life span and mass.

| **Common Name** | **Latin Name** | **Order** | **Family** | **MLS (yrs)** | **Mass (g)** | **Cad LD50** | **Cad S10_5** | **Cad S30_10** | **Perox LD50** | **Perox S10_5** | **Perox S30_10** |
| --- | --- | --- | --- | --- | --- | --- | --- | --- | --- | --- | --- |
| Northern Short-tailed Shrew | Blarinabrevicauda | Soricomorpha | Soricidae | 2 | 22 | 0.79 | 3.23 | 0.33 | 112 | 2.81 | 0.34 |
| Golden Hamster | Mesocricetusauratus | Rodentia | Muridae | 4 | 105 | 11.51 | 3.71 | 0.35 | >500 | 3.74 | 0.39 |
| Meadow Vole | Microtuspennsylvanicus | Rodentia | Cricetidae | 4 | 49 | 15.66 | 3.85 | 0.31 | 238 | 3.92 | 0.32 |
| Wild House Mouse | Musmusculus | Rodentia | Muridae | 4 | 20 | 0.97 | 2.87 | 0.36 | 85 | 2.54 | 0.34 |
| Cotton Rat | Sigmodonhispidus | Rodentia | Muridae | 5 | 185 | 0.67 | 3.40 | 0.38 | >2000 | 3.78 | 0.31 |
| Prairie Vole | Microtusochrogaster | Rodentia | Cricetidae | 5 | 40 | 0.46 | 2.15 | 0.32 | 61 | 0.71 | 0.48 |
| Gerbil | Merionesunguiculatus | Rodentia | Muridae | 6 | 53 | 22.68 | 3.37 | 0.36 | 799 | 3.25 | 0.29 |
| African Grass Rat | Arvicanthusniloticus | Rodentia | Muridae | 7 | 110 | 40.49 | 3.52 | 0.65 | 794 | 2.41 | 0.35 |
| Cactus Mouse | Peromyscuseremicus | Rodentia | Cricetidae | 7 | 25 | 0.17 | 2.84 | 0.38 | 40 | 2.07 | 0.51 |
| White-footed Mouse | Peromyscusleucopsis | Rodentia | Cricetidae | 8 | 23 | 2.20 | 2.69 | 0.42 | 198 | 2.93 | 0.47 |
| Deer Mouse (p.man) | Peromyscusmaniculatus | Rodentia | Cricetidae | 8 | 20 | 0.63 | 1.93 | 0.97 | 122 | 3.1 | 0.54 |
| Chipmunk | Tamiasstriatus | Rodentia | Sciuridae | 9 | 96 | 0.59 | 2.18 | 0.58 | 203 | 0.67 | 0.59 |
| Red Squirrel | Tamiasciurushudsonicus | Rodentia | Sciuridae | 10 | 200 | 44.19 | 2.05 | 0.74 | 84 | 2.81 | 0.56 |
| Guinea Pig | Caviaporcellus | Rodentia | Caviidae | 12 | 728 | 85.39 | 1.04 | 0.57 | >500 | 2.14 | 0.52 |
| Degu | Octodondegus | Rodentia | Octodontidae | 14 | 235 | 0.53 | 0.27 | 0.69 | 595 | 1.31 | 0.63 |
| Fox Squirrel | Sciurusniger | Rodentia | Sciuridae | 16 | 800 | 487.81 | 2.36 | 0.80 | >500 | 1.85 | 0.96 |
| Chinchilla | Chinchilla lanigera | Rodentia | Chinchillidae | 17 | 642 | 8.49 | 0.53 | 0.88 | 1271 | 0.59 | 1.10 |
| Big Brown Bat | Eptesicusfuscus | Chiroptera | Vespertilionidae | 34 | 23 | 66.62 | 0.48 | 2.22 | 327 | 0.87 | 0.81 |
| Raccoon | Procyonlotor | Carnivora | Procyonidae | 21 | 6000 | 45.79 | 0.85 | 1.12 | 155 | 1.17 | 1.32 |
| American Beaver | Castor canadensis | Rodentia | Castoridae | 23 | 20250 | 345.56 | 2.04 | 1.04 | 239 | 1.04 | 1.61 |
| North American Porcupine | Erethizondorsatum | Rodentia | Erethizontidae | 23 | 8600 | 22.63 | 2.70 | 1.35 | 77 | 1.35 | 1.69 |

**Supplemental Figure 1: Phylogenetic relationships among mammalian species**

The figure shows the phylogenetic tree used for the regression analyses shown in Figure 7. Time is in millions of years. Letters signify nodes used in the analysis. Note that branch lengths are not to scale, but have been adjusted for ease of reading. Phylogeny from ([Mercer & Roth 2003](#_ENREF_39); [Fabre *et al.* 2012](#_ENREF_18); [O'Leary *et al.* 2013](#_ENREF_44)).


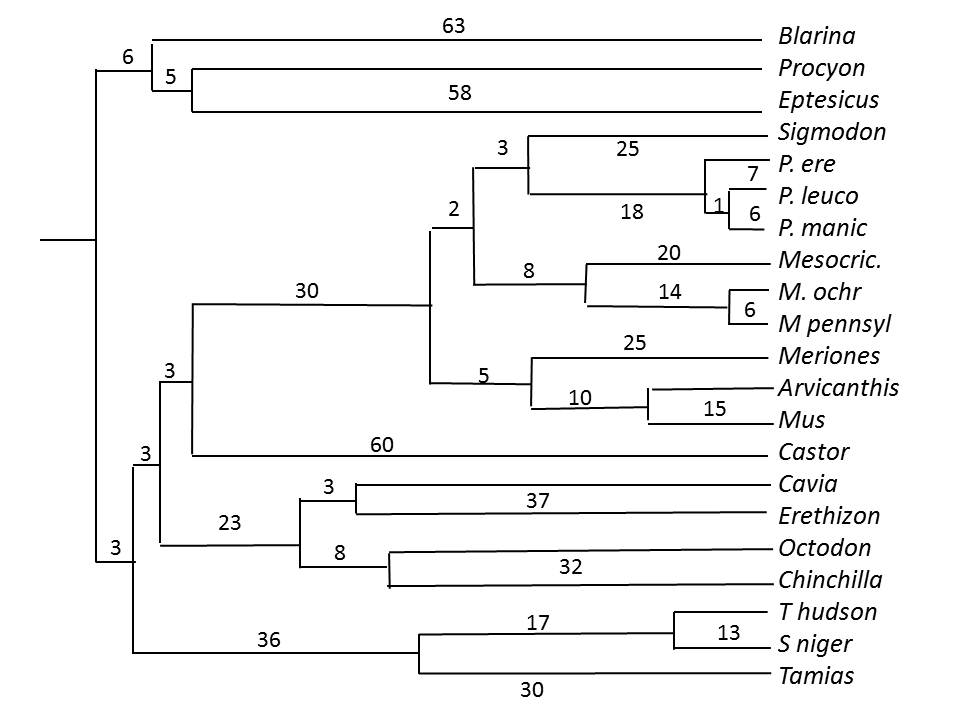


**Supplemental Figure 2: Phylogenetic relationships among avian species**

The figure shows the phylogenetic tree used for the regression analyses shown in Figure 7. Time is in millions of years. Letters signify nodes used in the analysis. Branch lengths not drawn to scale but have been adjusted for ease of reading. Phylogeny based on Jetz, et al. (2012).


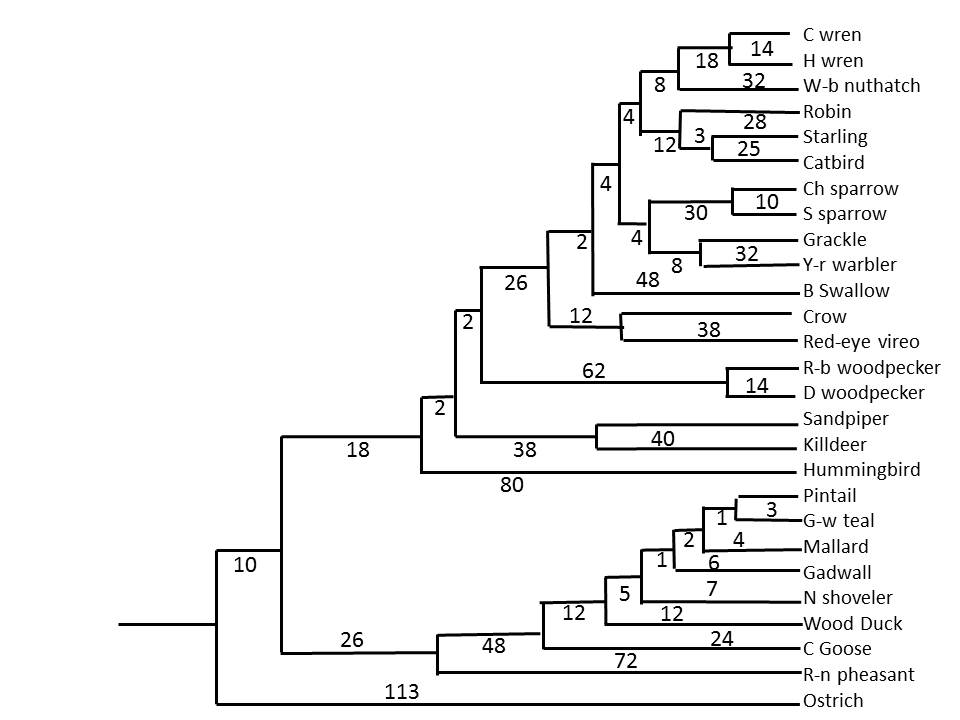

Supplement: Supplementary file 1 — Fig. S1 Phylogenetic relationships among mammalian species. Fig. S2 Phylogenetic relationships among avian species. Table S1 Taxonomy of birds used in this study, with maximum life span and mass. Table S2 Taxonomy of mammals used in this study, with maximum life span and mass. [file acel0013-0283-sd1.docx]
